# Coordinated In Vitro Release of Granulysin, Perforin and IFN-γ in TB and HIV/TB Co-Infection Associated with Clinical Outcomes before and after Anti-TB Treatment

**DOI:** 10.3390/pathogens9080655

**Published:** 2020-08-14

**Authors:** Nada Pitabut, Panadda Dhepakson, Shinsaku Sakurada, Naoto Keicho, Srisin Khusmith

**Affiliations:** 1 Faculty of Medicine, King Mongkut’s Institute of Technology Ladkrabang, Bangkok 10520, Thailand; nada.pi@kmitl.ac.th; 2 Medical Biotechnology Center, Medical Life Sciences Institute, Department of Medical Sciences, Ministry of Public Health, Nonthaburi 11000, Thailand; panadda.d@dmsc.mail.go.th; 3 Bureau of International Medical Cooperation, National Center for Global Health and Medicine, Tokyo 162-8655, Japan; ssakura@mti.biglobe.ne.jp; 4 Department of Pathophysiology and Host Defense, The Research Institute of Tuberculosis-Japan Anti-Tuberculosis Association, Tokyo 204-8533, Japan; nkeicho@jata.or.jp; 5 Department of Microbiology and Immunology, Faculty of Tropical Medicine, Mahidol University, Bangkok 10400, Thailand

**Keywords:** in vitro, perforin, granzyme-B, granulysin, IFN-γ, PPD, H37Ra, anti-TB treatment

## Abstract

Granule-associated killing molecules released from cytotoxic T lymphocytes participate as a crucial step in immunity against tuberculosis (TB), but the role of coordinated production remains controversial. Coordinated release of effector molecules in vitro after stimulating peripheral blood mononuclear cells (PBMCs) of active TB or HIV/TB coinfection patients with PPD, purified protein derivative of tuberculin and avirulent *Mtb*, H37Ra, an attenuated strain were investigated in association with clinical outcomes. Perforin, granzyme-B, granulysin and IFN-γ were measured using ELISA. Before anti-TB treatment, PBMCs of TB stimulated with PPD or H37Ra released higher perforin, granzyme-B, and granulysin levels than in HIV/TB and released significantly higher IFN-γ (*p* = 0.045, *p* = 0.022). Granulysin positively correlated with perforin in TB (*p* = 0.042, r = 0.385), HIV/TB coinfection (*p* = 0.003, r = 0.941) after PPD stimulation, and after H37Ra stimulation in TB (*p* = 0.005, r = 0.549), but negatively correlated with granzyme B in TB (*p* = 0.042, r = −0.386), HIV/TB coinfection (*p* = 0.042, r = 0.754) were noted. After anti-TB treatment, increased levels of perforin, granulysin and IFN-γ in TB or HIV/TB upon PPD or H37Ra stimulation, and decreased granzyme-B levels after PPD (*p* = 0.003) or H37Ra (*p* = 0.028) stimulation in TB were observed. These results suggest that granulysin may act synergistic with perforin and IFN-γ in TB, indicating its crucial function in host immunity to tuberculosis. Future studies with larger numbers of patients ought to be conducted in the future.

## 1. Introduction

Tuberculosis (TB) in humans can progress to active disease with various clinical manifestations in about 5–10% of people infected with *Mycobacterium tuberculosis* (*Mtb*). Worldwide, it is estimated that there were 10.4 million new TB cases and 480,000 new cases of multidrug-resistant TB (MDR-TB) causing 1.4 million deaths in 2015 [1].

The clinical outcome of pulmonary TB is divergent, ranging from complete pathogen clearance through asymptomatic latent infection to active TB, depending on the virulence of the pathogen, the effectiveness of anti-TB drugs, and host immune reactivity. Directing to the activation of the innate immune defense mechanism, alveolar macrophages and dendritic cells recognize and phagocytose *Mtb* which commences an intracellular signaling cascade, eliciting the adaptive immune response [2]. In persistent TB, the innate immune responses by macrophages and neutrophils are of critical importance [3]. Even though macrophages are armed with a wide battery of antimicrobial mechanisms, the control of bacterial proliferation and TB clinical progression is mainly depending on the activation signals delivered from CD4+ T cells [4] and CD8+ T cells [5].

IFN-γ plays a key role in CD4+ T cell-mediated macrophage activation in clearance of *Mtb* by inducing the expression of inducible nitric oxide synthase (iNOS) to enforce phagosome-lysosome fusion. The major source of IFN-γ during mycobacterial infection is T helper 1 (Th1)-polarized CD4+ T cells [6], however, other cytokines such as tumor necrosis factor alpha (TNF-α), interleukin (IL) -2 and IL-12 are likely to be included. More recently, a role of T helper lymphocytes secreting IL-17 (Th17 cells) has been identified [7,8]. Nevertheless, alteration in the balance in the cell-mediated immune response (CMI) reduces the protective effects against this granulomatous disease [9]. Thus, the activity of cytotoxic T lymphocytes (CTLs) towards *Mtb* inside macrophages is a critical step to reach full protection as well as to be cured of TB [10,11].

CTLs mediate apoptosis of infected target cells via granule- or receptor-mediated killing at the site of infection. CD8+ T cells and natural killer T cells (NKT cells) are the major cellular sources of perforin and granulysin which are the main cause of granule-mediated lysis of *Mtb* infected cells [12,13,14], while the target cells susceptibility to Fas/FasL-mediated killing depend on the contribution of Fas/FasL [15]. Functionally, CTLs produce and release cytolytic, e.g., perforin and granzymes and antimicrobial molecules, e.g., granulysin from cytolytic granules into intercellular synapse between the infected target cells and CTLs. Perforin induces the pore formation in target cell membranes facilitating delivery of granzyme and/or granulysin to the cytosol of target cells where they induce apoptosis via a recently identified membrane-repair mechanism [16]. However, it has been recently reported that Type 1 Treg cells (Tr1 cells) induced apoptosis via granulysin/granzyme B, not via perforin [17]. In human, granzymes A and B are translocated rapidly to the nucleus inducing the apoptosis of target cells [18], whereas granulysin can directly trigger apoptosis without cell contact [19] and attack the mycobacteria cell membrane in the endosomes of macrophages [14]. Granulysin penetrates firstly into infected cells and releases bactericidal granzymes into bacteria intracytoplasmic membranes [20]. Thus, in the presence of granulysin, granzymes mediate bacterial cell death independently of host cell death [21] by creating cell wall lesions which promote osmotic lysis of *Mtb* in a perforin-dependent manner [9]. Therefore, the coordinated expression of granulysin and perforin might be one of the extensive host immune defense mechanisms to control TB [22,23]. Mechanistically, coexpression of perforin and granulysin, however, led to efficient killing of intracellular *Mtb*, defining a clear role for perforin in providing access of antimicrobial granule proteins to their targets. The perforin dependency of granulysin was confirmed in a model of *Listeria innocua* infection of dendritic cells [20].

Significantly increased IFN-γ levels were observed in vitro in stimulated peripheral blood mononuclear cells (PBMCs) with purified protein derivative (PPD) in TB patients and healthy controls compared to unstimulated PBMCs [24], suggesting the major role of Th1 cytokines during the host response to *Mtb* [6]. IFN-γ released from H37Rv stimulated PBMCs from HIV/TB with immune reconstitution inflammatory syndrome (IRIS), and increased granzyme-B production from HIV/TB without IRIS in comparison with unstimulated PBMCs [25]. However, the intracellular granzyme-B expression by specific lymphocyte populations and the elevated serum levels in patients with active pulmonary TB indicate the host defense against *Mtb* [26].

In an in vitro model, it has become apparent that the effective defense against TB is dependent on the protective immune response of the host and the potential of *Mtb* strains to evade or induce the host defense mechanisms [27,28]. In mouse model, the virulent *Mtb* H37Rv strain and avirulent *Mtb* H37Ra strain, derived originally from the clinical virulent strain H37, are differentiate by their abilities to cause progressive disease [2]. However, it is noted that *Mtb* H37Ra is regarded as an attenuated strain rather than being completely avirulent because considerable bacterial growth was observed in macrophages in vitro [29]. In addition, *Mtb* H37Ra could be used to assess the possible confounding factors in infection models and to characterize key elements in the early human host immune response to *Mtb* infection [30]. Moreover, the release of perforin, granzyme B, granulysin and IFN-γ from effector cells may play a major role in host immune mechanisms efficient in restricting the growth of *Mtb*. In this study, the coordinated release of these effector molecules by PBMCs from active TB or HIV/TB coinfected patients after in vitro stimulation with PPD or avirulent *Mtb*, H37Ra was investigated in association with clinical outcomes before and after anti-TB treatment.

## 2. Results

### 2.1. Higher Releases of In Vitro Perforin, Granzyme-B, Granulysin and IFN-γ from Stimulated PBMCs with PPD and H37Ra in Active TB Patients before and after Anti-TB Treatment

**Figure 1:**
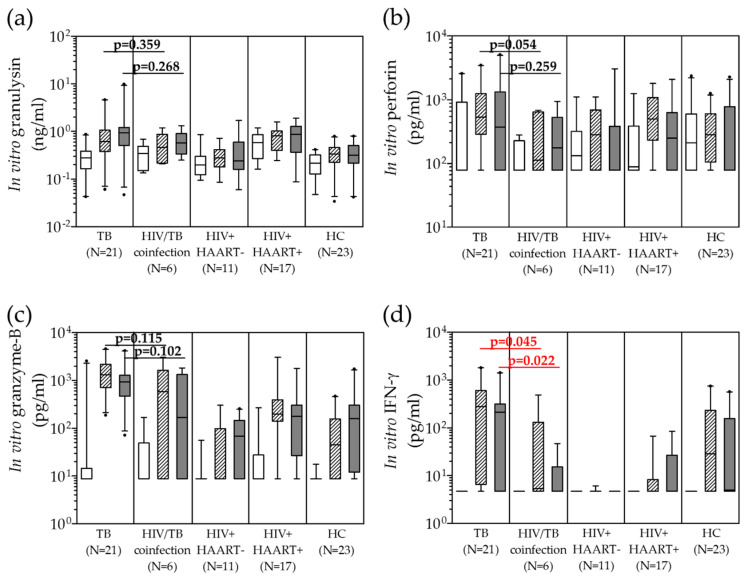
Distribution of in vitro release of granulysin (**a**), perforin (**b**), granzyme-B (**c**) and IFN-γ (**d**) from peripheral blood mononuclear cells stimulated with PPD (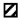) and H37Ra (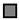) in comparison with unstimulated PBMCs (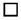) of TB, HIV/TB coinfection, HIV+HAART−, HIV+HAART+ and healthy controls before anti-TB treatment. A horizontal bar indicated the median of each group. Statistical significant by Mann Whitney U test, *p* < 0.05. (PPD = purified protein derivative, H37Ra = avirulent stain heat killed *Mtb*; HIV+HAART− = HIV patients without receiving highly active antiretroviral therapy (HAART); and HIV+HAART+ = HIV patients receiving HAART.

Before anti-TB treatment, the in vitro levels of granulysin, perforin, granzyme-B and IFN-γ released from PBMCs of active TB or HIV/TB coinfection patients after PPD and H37Ra stimulation were analyzed in comparison with those of HIV+HAART−, HIV+HAART+ and healthy individuals (HC) as controls (Table 1). Upon stimulation, the non-significantly higher median levels in TB than HIV/TB coinfection were observed in granulysin (Figure 1a), perforin (Figure 1b) and granzyme-B (Figure 1c). However, significantly different IFN-γ levels were evident after PPD and H37Ra stimulation in TB or HIV/TB coinfection (Figure 1d).

**Figure 2:**
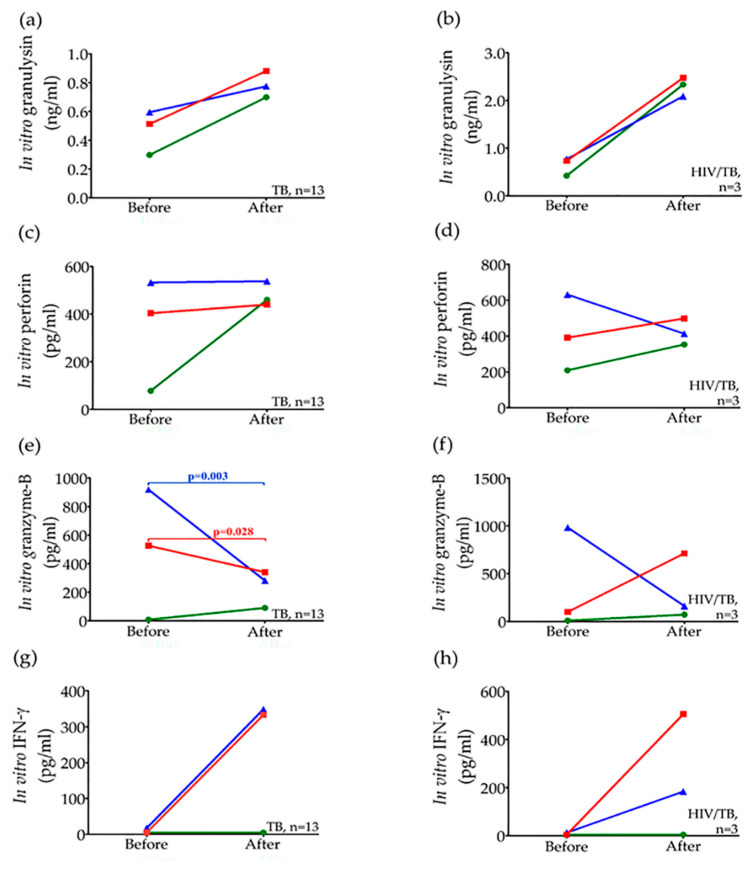
Alteration of in vitro granulysin (**a**,**b**), perforin (**c**,**d**), granzyme-B (**e**,**f**) and IFN-γ (**g**,**h**) levels from stimulated peripheral blood mononuclear cells with PPD (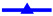) and H37Ra (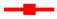) in comparison with unstimulated (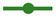) in active TB and HIV/TB coinfection before and after anti-TB treatment. Wilcoxon Signed Rank test was used to compare different levels before and after completion of anti-TB treatment. *p* value < 0.05 was statistical significant.

After treatment for 6–9 months considered as cured, the altered release of these effective molecules from PBMCs of TB and HIV/TB coinfection after PPD or H37Ra stimulation are shown in Figure 2. The median granulysin and IFN-γ levels tended to increase after treatment (TB, PPD: before treatment, 0.595 ng/mL; after treatment, 0.775 ng/mL, *p* = 0.311, and H37Ra: before treatment, 0.513 ng/mL; after treatment, 0.881 ng/mL, *p* = 0.507, Figure 2a). Similar observations were found in the circulating granulysin levels with significant difference in TB patients and HIV/TB coinfected patients (Table 2). Whereas the median perforin levels after H37Ra stimulation were increased in both TB (before treatment, 404 pg/mL; after treatment, 440 pg/mL, *p* = 0.912, Figure 2c) and HIV/TB coinfection (before treatment, 391 pg/mL; after treatment, 498 pg/mL, *p* = 0.593, Figure 2d). However, after PPD stimulation, there was no change in perforin levels in TB (before treatment, 533 pg/mL; after treatment, 538 pg/mL, *p* = 0.780, Figure 2c), but decrease in HIV/TB coinfection (before treatment, 631 pg/mL; after treatment, 413 pg/mL, *p* = 1.000, Figure 2d). These results were consistent with the increased circulating perforin levels after treatment in active TB patients and HIV/TB coinfected patients (Table 2). Obviously, after completion of treatment, the median granzyme-B levels after PPD or H37Ra stimulation of PBMCs significantly decreased in TB (PPD: before treatment, 919.33 pg/mL; after treatment, 282.0 pg/mL, *p* = 0.003 and H37Ra: before treatment, 527 pg/mL; after treatment, 431 pg/mL, *p* = 0.028) (Figure 2e), tended to decrease after PPD stimulation in HIV/TB coinfection (before treatment, 982.67 pg/mL; after treatment, 161.0 pg/mL, *p* = 0.285), and increased after H37Ra stimulation (before treatment, 99.33 pg/mL; after treatment, 713.0 pg/mL, *p* = 0.593) (Figure 2f). However, these in vitro levels appear to contradict the increased circulating granzyme-B levels after treatment with significance in active TB, and non-significance in HIV/TB coinfection (Table 2).

In addition, IFN-γ released from PBMCs of TB and HIV/TB coinfection after in vitro PPD and H37Ra stimulation tended to increase after treatment (TB, PPD: before treatment, 17.32 pg/mL; after treatment, 349.51 pg/mL, *p* = 0.917, and H37Ra: before treatment, <4.7 pg/mL; after treatment, 333.57 pg/mL, *p* = 0.110, (Figure 2g) (HIV/TB coinfection, PPD: before treatment, 12.79 pg/mL; after treatment, 184.0 pg/mL, *p* = 1.00, and H37Ra: before treatment, <4.7 pg/mL; after treatment, 505.79 pg/mL, *p* = 0.285 (Figure 2h). Conversely, the circulating IFN-γ levels in TB and HIV/TB coinfection patients decreased after treatment (Table 2).

### 2.2. Significant Correlation of Granulysin, Perforin and Granzyme-B Levels from Stimulated PBMCs in Active TB and HIV/TB Coinfection before Anti-TB Treatment

**Figure 3:**
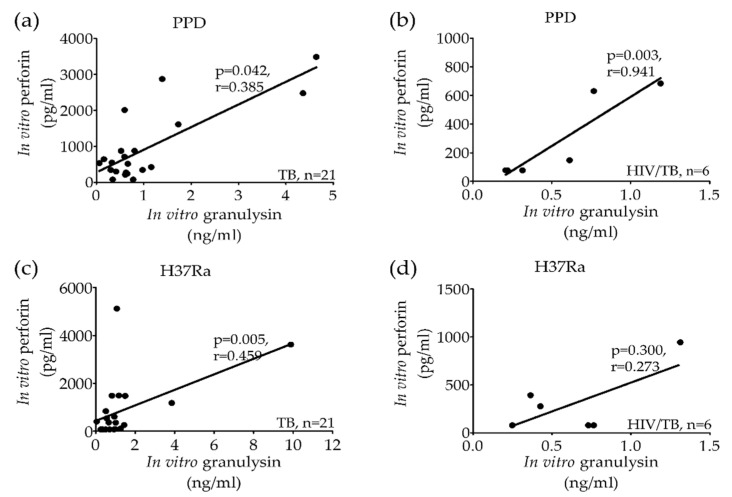

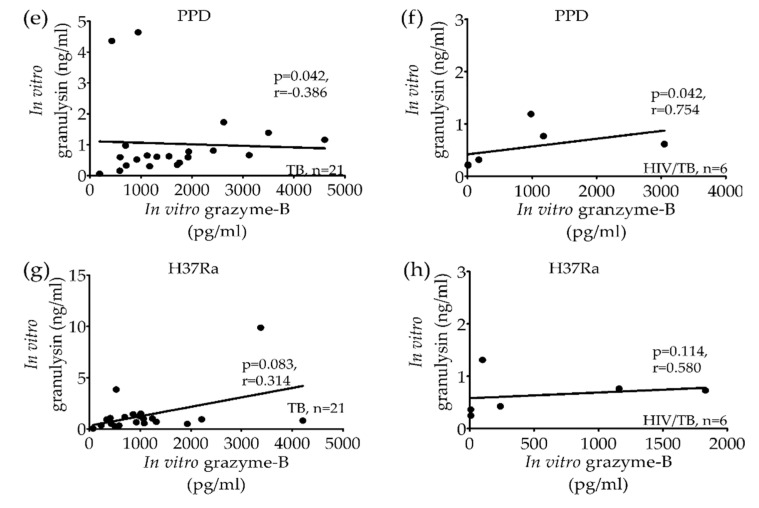
Correlation of in vitro granulysin, perforin and granzyme-B levels after PPD and H37Ra stimulation of peripheral blood mononuclear cells of active TB (**a,c,e,g**) and HIV/TB coinfection (**b,d,f,h**) before anti-TB treatment. Correlations were assessed using Spearman’s rank correlation test (*p* < 0.05).

To evaluate the host defense mechanism of granule exocytosis before anti-TB treatment, the released levels of perforin, granzyme-B and granulysin from stimulated PBMCs of active TB or HIV/TB coinfection with PPD or H37Ra were correlated. After PPD stimulation, the granulysin levels was correlated significantly with perforin in TB (Figure 3a) and HIV/TB coinfection (Figure 3b). Whereas after H37Ra stimulation, the levels of granulysin and perforin were significantly correlated in TB (Figure 3c), but not in HIV/TB coinfection (Figure 3d).

Interestingly, after PPD stimulation, the granulysin levels were significantly correlated with granzyme-B in TB (Figure 3e) and HIV/TB coinfection, indicating an inverse correlation (Figure 3f), while upon H37Ra stimulation, non-significant correlations were found in both TB (Figure 3g) and HIV/TB coinfection (Figure 3h).

## 3. Discussion

In this study, two major cytolytic proteins of lytic granules eg. perforin and granzymes and antimicrobial molecules, e.g., granulysin, as well as IFN-γ were upregulated after in vitro PPD or H37Ra stimulation in active TB compared to HIV/TB coinfection before anti-TB treatment which increased after treatment, indicating the importance of these effector molecules in host immune mechanism against tuberculosis. Granulysin may act synergistically with perforin and IFN-γ by the significant correlation of granulysin with granzyme-B and perforin levels in TB or HIV/TB coinfection, and their effector mechanisms could contribute to control TB. These in vitro results support the existence of the elevated circulating granulysin in relation to perforin levels in clinical TB and HIV/TB coinfected patients in this study as well as those of previous report [31], confirming the role of granule exocytosis in host immune mechanism. However, perforin and granzyme-B have been shown to co-express with granulysin [32] and were upregulated by CD4+ T cells after in vitro H37Ra restimulation [33]. An induction of perforin, granzyme-B and granulysin expressions in memory CD4+ T cells in TB principally indicated the granule-exocytosis mechanism rather than the CD95-dependent apoptotic pathway for target cell killing [32,33]. For PPD-positive individuals, the coordinated expression of granulysin, perforin, and granzyme-B in CD3+ T cells correlated with the control of *Mtb* infection, and increased mRNA expression of these molecules were associated with the inhibition of mycobacterial growth in macrophages [34]. Although CD8+ T cells expressing granulysin and perforin were scarce in granuloma lesions in TB [9], however, perforin is critical for host immune defense during *Mtb* infection, even though the cytolytic activity in vivo is slightly affected in the absence of perforin [35]. Thus, the increasing released perforin levels after cured in PBMCs of active TB upon H37Ra stimulation confirmed its effective role against *Mtb* infection.

Similar to the in vitro findings, before anti-TB treatment, high circulating perforin, granzyme B and granulysin levels in sera of TB and HIV/TB coinfection patients were seen, which increased after treatment, presumably indicating its role of granule exocytosis, except for IFN-γ which decreased after treatment with cure, indicating host defense against *Mtb* infection. These results are agreeable with the findings previously shown of circulating IFN-γ and granulysin before and after completion of treatment [31], as well as the correlation of granulysin and cellular IFN-γ production with curative host responses in pulmonary TB, pointing to the potential role of granulysin, beside IFN-γ, in defense mechanisms in adults and children [32]. In addition, granulysin with diverse activities of NK cells and CTL in physiological and pathological settings in viral infection could be a useful novel serum marker to evaluate the overall status of host cellular immunity [36]. These results provide evidence that these effector molecules could be potential predictive biomarkers for immune status of TB [32].

Obviously, granulysin seems to be more of an effector molecule during initiation of *Mtb* infection by the negative correlation of granulysin and granzyme-B releases from PBMCs of active TB after PPD stimulation before treatment. However, the trend seems to be driven by two patients/data points only. In addition, due to limitation of sample size of HIV/TB coinfection, granulysin correlation driven by a single patient/data point for HIV/TB confection is too low to take any conclusion. It need more sample size for further study in future. The significant reduction after treatment of granzyme-B from PBMCs of active TB after PPD or H37Ra stimulation suggested its role in host defense mechanism. The same phenomenon after treatment was seen in PBMCs of HIV/TB coinfection upon PPD stimulation. However, variable results after H37Ra stimulation were noted in which one unchanged, one increased and one decreased. This discrepancy cannot be concluded due to the limited number of sample sizes in which PBMCs from only three HIV/TB coinfected patients could be obtained during the study period because of the patients’ death.

The opposing results seen in granzyme-B after treatment were the decrease of in vitro granzyme-B from PBMCs of TB after PPD or H37Ra stimulation and the significant increase of circulating granzyme-B levels in TB patients’ sera, implying the impaired cytolytic molecules during active TB as well as immune-escaping mechanisms of *Mtb*. These observed discrepancies could be possibly due to (i) granzyme-B, one of granule exocytosis molecules might be utilized during the continuous immune effector mechanism in vivo for controlling *Mtb* infection; (ii) more granzyme-B released in the supernatant might possibly be from primed T cells containing in PBMCs upon in vitro re-stimulation with PPD or H37Ra; and (iii) granzyme-B was determined as an extracellular molecule outside the cells in supernatant, so how much granzyme-B resides in the cells or releases outside is questionable. This was not surprising since many variable results have been previously demonstrated. It is suggested that granzyme-B levels might not be the best surrogate for CTLs since no correlation of the moderate decreased granzyme-B and the reduced in vivo killing could be demonstrated [34]. While another in vitro study revealed that after stimulation with *Mtb* antigens including H37Ra, both CD8+ and CD4+ T cells upregulated the mRNA expression of granulysin, perforin, granzyme-A and B and CD95L (Fas ligand), enable to lyse *Mtb* infected target cells by mediated mainly via the granule exocytosis pathway [35]. In TB patients, a slightly increased expression of granzyme-B was shown in CD8+ and CD4+ T cells, but not NK cells [26,37], which is contradict to the recent findings that active pulmonary TB patients downregulated granzyme-B expression in CD8+ T cells in comparison with latent TB infection [38]. Therefore, larger sample sizes are essential to gain better understanding the dynamics of these cytolytic proteins.

IFN-γ, primarily secreted by activated macrophages, NK cells and T cells, is crucial for macrophage activation and antigen presentation, as well as promoting cell proliferation, cell-cell adhesion and apoptosis. It is noted that PBMCs of TB patients upon PPD or H37Ra stimulation released significantly higher IFN-γ levels than those of HIV/TB coinfection before treatment, with greater increase after completion of treatment, indicating their ability to induce Th1 cytokine production, a critical mediator of host defense against *Mtb* infection. Our results support the recent findings on the higher IFN-γ production by stimulated PBMCs with PPD in patients with infiltrative TB compared to other clinical features of TB and healthy controls [24]. Moreover, the increased IFN-γ production from PBMCs of pulmonary TB patients upon stimulation with either early secretory antigenic target of 6 kDa (ESAT6) or culture filtrated protein 10 (CFP10), and the high expression of IFN-γ by CD4+ T cells and CD8+ T cells have been recently demonstrated [39].

## 4. Materials and Methods 

### 4.1. Ethical Statement

Ethical approval to conduct this study was obtained from the Ethical Review Committee for Research in Human Subjects, Ministry of Public Health, Thailand (Approval Number:15/2550). Written informed consents were obtained from all studied participants.

### 4.2. Peripheral Blood Mononuclear Cells (PBMCs)

PBMCs isolated from whole blood in K_3_EDTA (3 mL) (Greiner Bio-One, Bangkok, Thailand) from 21 patients with active TB and six with HIV/TB coinfection without receiving highly active antiretroviral therapy (HAART) before and after 6–9 months of anti-TB treatment considered as cured were included in this study (13 TB and three HIV/TB coinfection). PBMCs from 11 HIV patients without HAART (HIV+HAART−), 17 HIV patients receiving HAART (HIV+HAART+) and 23 healthy individuals (HC) were used as controls.

PBMCs were isolated by standard Ficoll-Hypaque gradient centrifugation. Briefly, an equal volume of whole blood (3 mL) in K_3_EDTA diluted phosphate buffer saline (PBS) was layered over Ficoll-paque PLUS (3 mL) (Amersham Biosciences, Uppsala, Sweden). After centrifugation at 1000× *g* for 20 min at room temperature, PBMCs were harvested and washed twice with incomplete RPMI 1640 medium (Gibco, Invitrogen, Grand Island, NY, USA) by centrifugation at 700× *g* for 10 min at 4 ^o^C. The pellets were suspended in cold RPMI 1640 complete medium containing 10% fetal bovine serum (FBS) (Gibco, Invitrogen). The viable PBMCs were evaluated using 0.2% Trypan blue dye.

### 4.3. Stimulation of PBMCs with Purified Protein Derivative (PPD) and Avirulent Heat Killed Mtb (H37Ra) Strain

Approximately 1 × 10^6^ PBMCs in 1 mL of complete RPMI 1640 and 2-mercaptoethanol (2-ME) (Gibco, Invitrogen) were plated in 24 well plates (Costar, Tewksbury, MA, USA). The test was done in duplicate. The cells were stimulated with 20 µg/mL of PPD (Japan BCG laboratory, Tokyo, Japan) or H37Ra strains (Difco, Detroit, MI, USA) by incubation at 37 °C in 5% CO_2_ [40] for 40 h. The granulysin, perforin, granzyme-B and IFN-γ released in the supernatants were measured. Unstimulated PBMCs and stimulated PBMCs with PPD were used as negative and positive controls, respectively.

### 4.4. Determination of Granulysin, Perforin, Granzyme-B and IFN-γ Releases from Stimulated PBMCs In Vitro

The granulysin, perforin, granzyme and IFN-γ release from stimulated PBMCs with PPD and H37Ra were measured by ELISA [31,40]. Plates (Costar) were coated with 5 µg/mL of mouse RB1 monoclonal antibody (mAb) (MBL International Corporation, Nagoya, Japan) and incubated overnight at 4 °C. The undiluted supernatants from stimulated PBMCs with PPD or H37Ra and unstimulated PBMCs were added and incubated for 2 h at room temperature. The detection of bound antigen were done with 0.1 µg/mL of mouse RC8 mAb (MBL International Corporation) and avidin-horseradish peroxidase (Av-HRP) conjugate (BD Biosciences Phamingen, San Diego, CA, USA). The color was developed by tetramethylbenzidine (TMB) substrate (BD Biosciences Pharmingen). The optical density was measured at 450/570 nm using microplate reader (Sunrise; Tecan, Männedorf, Switzerland). Culture supernatant from Cos7 cell transfected with gene encoding 15 kDa granulysin were used for standard curve for granulysin concentration (kindly provided by Dr. Masaji Okada, Kinki-Chuo Chest Medical Center, Osaka, Japan). The low limit of granulysin detection is 0.047 ng/mL.

The perforin, granzyme-B and IFN-γ releases were measured from stimulated PBMCs with PPD or H37Ra by ELISA according to the manufacture protocols (MABTECH AB, Stockholms, Sweden for perforin and granzyme-B, and BD Biosciences Pharmingen for IFN-γ). The low limits of detection for perforin, granzyme-B and IFN-γ assays were 78, 8.78 and 4.7 pg/mL, respectively.

### 4.5. Statistical Analyses

Statistical analyses using SPSS software version 18.0 (SPSS, Inc., Chicago, IL, USA) were performed. Kruskal-Wallis test was used for multiple comparisons to compare perforin, granzyme-B, granulysin and IFN-γ among subject groups, while Mann-Whitney U test was used for two independent subject groups. Wilcoxon signed-rank test was used to compare different levels of effector molecules before and after anti-TB drug treatment. Correlations were assessed using Spearman’s rank correlation test. *p* value < 0.05 is statistically significant.

## 5. Conclusions

Insights into the coordinated production of cytolytic effector molecules and cytokines by effector T cells which participate as a crucial step in protective immunity against tuberculosis are needed to tailor control measures. The alterations of granulysin coordinated with perforin and IFN-γ increased after anti-TB treatment in active TB and HIV/TB coinfection. Granulysin may act synergistically with perforin and IFN-γ, pointing to its critical importance for the control of tuberculosis. These findings provide evidence suggesting that granulysin, perforin and IFN-γ may serve as immune markers for prediction of active TB, and their changes would be the prognostic markers for therapeutic efficacy. Future studies with larger numbers of patients ought to be conducted in the future.

## Figures and Tables

**Table 1:** In vitro released levels of granulysin, perforin, granzyme-B and IFN-γ from peripheral blood mononuclear cells (PBMCs) stimulated with PPD and H37Ra in active TB, HIV/TB coinfection, HIV+HAART−, HIV+HAART+ and healthy individuals before anti-TB treatment.

Measurement	Stimulation with	PBMCs from				
		Active TB ^1^ (*n* = 21)	HIV/TB Coinfection ^2^ (*n* = 6)	HIV+ HAART− ^3^ (*n* = 11)	HIV+ HAART+ ^4^ (*n* = 17)	HC ^5^ (*n* = 23)
Granulysin (ng/mL) (median, IQR)	Medium alone	0.281, 0.166–0.389	0.346, 0.149–0.488	0.197, 0.123–0.300	0.584, 0.271–0.856	0.217, 0.127–0.320
	PPD ^6^	0.623, 0.380–1.066	0.464, 0.217–0.872	0.278, 0.180–0.417	0.817, 0.397–1.026	0.334, 0.226–0.460
	H37Ra ^7^	0.931, 0.509–1.218	0.578, 0.335–0.901	0.243, 0.159–0.595	0.864, 0.365–1.269	0.315, 0.216–0.505
Perforin (pg/mL) (median, IQR)	Medium alone	<78, <78–918.5	<78, <78–227	132, <78–321	89, <78–386	210, <78–593
	PPD ^6^	533, 286–1246	113, <78–644	284, <78–694	499, 231–1075	283, 105–605
	H37Ra ^7^	370, <78–1330	177, <78–529	78, <78–384	249, <78–630	<78, <78–783
Granzyme-B (pg/mL) (median, IQR)	Medium alone	<8.79, <8.79–14.40	<8.79, <8.79–49.01	<8.79	<8.79, <8.79–27.5	<8.79
	PPD ^6^	1313, 704–2178	587.7, <8.79–1644.5	<8.79, <8.79–98.33	197, 140.7–395.3	44.67, <8.79–156.3
	H37Ra ^7^	924.67, 467.33–1279	168.7, <8.79–1327.4	67.67, <8.79–146	176.67, 26.89–305.83	159, 12.0–306.33
IFN-γ (pg/mL) (median, IQR)	Medium alone	<4.7	<4.7	<4.7	<4.7	<4.7
	PPD ^6^	279.25, 6.57–607.5	5.4, <4.7–131.34	<4.7	<4.7, <4.7–8.245	28.6, <4.7–230.73
	H37Ra ^7^	211.85, <4.7–313.1	<4.7, <4.7–15.30	<4.7	<4.7, <4.7–26.98	5.04, <4.7–156.51

^1^ Active TB = PBMCs from TB patients; ^2^ HIV/TB coinfection = PBMCs from HIV/TB co-infected patients without receiving highly active antiretroviral therapy (HAART); ^3^ HIV+HAART− = PBMCs from HIV patients without receiving HAART; ^4^ HIV+HAART+ = PBMCs from HIV patients receiving HAART; ^5^ HC = PBMCs from healthy individuals without stimulation as negative controls; ^6^ PPD = purified protein derivative; ^7^ H37Ra = avirulent stain heat killed *Mtb.*

**Table 2:** Circulating granulysin, perforin, granzyme-B and IFN-γ levels in patients with active TB and HIV/TB coinfection before and after anti-TB treatment whose peripheral blood mononuclear cells were obtained.

Circulating Concentration (Median, IQR)	Active TB (*n* = 13)		*p*-Value ^1^	HIV/TB Coinfection (*n* = 3)		*p*-Value ^1^
	Before Treatment	After Treatment		Before Treatment	After Treatment	
* Granulysin **(ng/mL)**	1.050 (0.635–1.350)	1.860 (1.190–4.645)	**0.007**	7.37 (3.75–10.07)	9.50 (8.54–10.24)	0.109
**Perforin** **(pg/mL)**	5538, (4243–8480)	8575, (7195–12,980)	**0.001**	10,763, (10,305–13,255)	13,865, (11,315–19,290)	0.285
**Granzyme-B** **(pg/mL)**	<8.79, <8.79–31.0	118, 56.67–145.5	**0.006**	26, <8.79–41.33	83.33, 71–171.33	0.109
* IFN-γ **(pg/mL)**	10.04 (<4.70–21.12)	5.04 (<4.7–8.435)	0.139	53.04 (6.72–89.54)	<4.7 (<4.7–15.08)	0.285

^1^ Statistically significant differences indicated by a Wilcoxon Signed Rank test (*p*-value < 0.05); * Circulating granulysin (ng/mL) and IFN-γ (pg/mL) [30].
